# Facile and Scalable Preparation of Graphene Oxide-Based Magnetic Hybrids for Fast and Highly Efficient Removal of Organic Dyes

**DOI:** 10.1038/srep12451

**Published:** 2015-07-29

**Authors:** Tifeng Jiao, Yazhou Liu, Yitian Wu, Qingrui Zhang, Xuehai Yan, Faming Gao, Adam J. P. Bauer, Jianzhao Liu, Tingying Zeng, Bingbing Li

**Affiliations:** 1 State Key Laboratory of Metastable Materials Science and Technology, Yanshan University, Qinhuangdao 066004, P. R. China; 2 Hebei Key Laboratory of Applied Chemistry, School of Environmental and Chemical Engineering, Yanshan University, Qinhuangdao 066004, P. R. China; 3 Department of Chemistry and Biochemistry, Central Michigan University, Mount Pleasant, MI 48859, USA; 4 National Key Laboratory of Biochemical Engineering, Institute of Process Engineering, Chinese Academy of Sciences, Beijing 100190, P. R. China; 5 Research Laboratory for Electronics, Massachusetts Institute of Technology, Cambridge, MA 02139, USA

## Abstract

This study reports the facile preparation and the dye removal efficiency of nanohybrids composed of graphene oxide (GO) and Fe_3_O_4_ nanoparticles with various geometrical structures. In comparison to previously reported GO/Fe_3_O_4_ composites prepared through the one-pot, *in situ* deposition of Fe_3_O_4_ nanoparticles, the GO/Fe_3_O_4_ nanohybrids reported here were obtained by taking advantage of the physical affinities between sulfonated GO and Fe_3_O_4_ nanoparticles, which allows tuning the dimensions and geometries of Fe_3_O_4_ nanoparticles in order to decrease their contact area with GO, while still maintaining the magnetic properties of the nanohybrids for easy separation and adsorbent recycling. Both the as-prepared and regenerated nanohybrids demonstrate a nearly 100% removal rate for methylene blue and an impressively high removal rate for Rhodamine B. This study provides new insights into the facile and controllable industrial scale fabrication of safe and highly efficient GO-based adsorbents for dye or other organic pollutants in a wide range of environmental-related applications.

Extensive effort has been made to introduce permanently anchored magnetic nanoparticles to graphene oxide (GO) or reduced GO (rGO) sheets^1^,^2^,^3^,^4^,^5^,^6^. For instance, O. Akhavan *et al*., reported the successful preparation and the magnetic separation application of superparamagnetic ZnFe_2_O_4_/reduced graphene oxide (rGO) composites by hydrothermal reaction method^5^. In general, magnetic nanocomposites are prepared through the *in situ* deposition of magnetic nanoparticles (i.e., Fe_3_O_4_ nanoparticles) by co-precipitating iron salts onto GO/rGO sheets in aqueous solution^1^,^2^,^3^,^4^. Chemically anchoring Fe_3_O_4_ nanoparticles on the GO/rGO sheets allows higher loading and uniform distribution of these nanoparticles, giving rise to magnetic composites that possess interesting electrochemical and magnetic properties^1^,^2^,^3^,^4^,^5^,^6^. The rGO/Fe_3_O_4_ composite containing permanently anchored Fe_3_O_4_ nanoparticles has also been evaluated for its dye adsorption and removal capacity^2^,^3^. These studies demonstrate the contribution of anchored Fe_3_O_4_ nanoparticles to the composite’s magnetic properties, which allow easy magnetic separation of dye-adsorbed composites from water. However, the dye removal capacity of these materials solely depends on the available surface area of GO or rGO sheets (i.e., the available surface area for π-π stacking interactions) along with abundant functional groups on the GO sheets^2^,^7^,^8^. Consequently, the adsorption capacities of pristine GO or rGO sheets can be compromised due to the permanently anchored Fe_3_O_4_ nanoparticles^2^,^3^. Particularly, the Fe_3_O_4_ nanoparticles grown through *in situ* deposition are ultrafine nanoparticles (e.g., less than 50 nm diameter), which can occupy a greater surface area than relatively larger nanoparticles of the same total mass. To overcome the above drawbacks of the *in situ* grown, permanently anchored Fe_3_O_4_ nanoparticles, the facile preparation of GO/Fe_3_O_4_ hybrids reported in this study was achieved by combining GO and Fe_3_O_4_ nanoparticles through physical affinities between sulfonated GO and Fe_3_O_4_ nanoparticles. Benefiting from the larger aspect ratio of the GO sheets used in this study, the Fe_3_O_4_ nanoparticles can also be easily wrapped in the GO sheets, further assisting in the magnetic separation. In addition, the Fe_3_O_4_ nanostructures were prepared separately and various dimensions and geometries can be conveniently obtained, allowing us to further explore the effects of the Fe_3_O_4_ nanostructures’ dimensions and geometries on the dye adsorption capacity of GO sheets. Overall, the affordable access to Fe_3_O_4_ nanostructures and GO sheets allow the reported facile preparation procedure to be adopted in various laboratory conditions and to be further optimized for large-scale manufacturing of green and safe dye adsorbents.

## Results and Discussion

**Figure 1:**
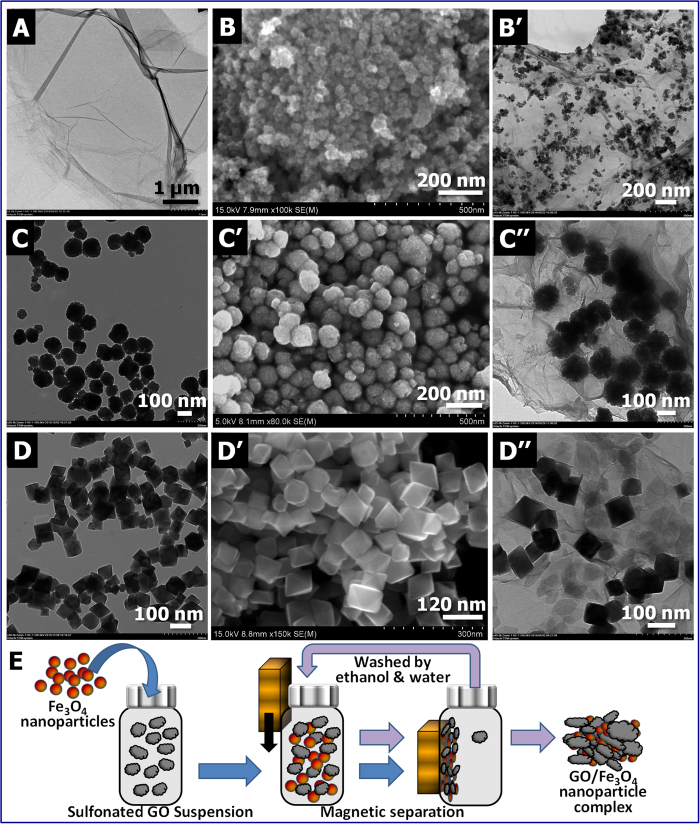
(**A**) A TEM image of a GO sheet. Morphology of Fe_3_O_4_ nanoparticles includes (**B**) fine nanoparticles (<100 nm diameter), (**C**, **C**’) nanospheres (~100 nm diameter), and (**D**, **D**’) multifaceted nanoparticles. The nanohybrids shown in (**B**’, **C**”, and **D**”) were prepared with Fe_3_O_4_ nanoparticles seen in (**B**, **C**’, and **D**’), respectively, using 5:4 by mass GO/Fe_3_O_4_ (i.e., G5F4). [Note: TEM images: **A**, **B**’, **C**, **C**”, **D**, and **D**”, SEM images: **B**, **C**’ and **D**’].

In this study, GO nanostructures were prepared according to a modified Hummer’s method^9^,^10^. The GO sheets were then sulfonated to enhance their surface affinity to Fe_3_O_4_ nanoparticles^11^,^12^. A typical TEM image of GO nanostructure is shown in Fig. 1A. Magnetic Fe_3_O_4_ nanoparticles with well-defined geometries were prepared separately using a modified solvothermal method^13^. Detailed experimental procedures are included in Methods and are briefly described as follows: sodium hydroxide (NaOH, 0.4 g, 0.6 g, or 0.8 g) and 1.0 g of polyethylene glycol (PEG, M_w_ ~ 6000 g·mol^−1^) was added to a solution containing 1.35 g of ferric chloride hexahydrate (FeCl_3_∙6H_2_O) and 30 mL of ethylene glycol (C_2_H_6_O_2_). The above solution mixture was stirred for 30 minutes, transferred into a Teflon-lined stainless steel autoclave (~100 mL), and then allowed to react for 8 hours at 200 °C. After the reaction was complete, a black precipitate (i.e., Fe_3_O_4_ nanoparticles) was collected with a magnetic block, purified by alternatively washing several times with deionized water and ethanol, and dried at 60 °C in a vacuum oven. The geometry of the Fe_3_O_4_ nanoparticles was found to vary with the initial mass of NaOH. For instance, when the above synthesis route started with 0.4 g of NaOH, the as-prepared Fe_3_O_4_ nanoparticles were less than 100 nm in diameter (Fig. 1B). In contrast, increasing the initial mass of NaOH to 0.6 g gave rise to nanospheres with a diameter above 100 nm, as shown in Fig. 1C,C’. Further increasing the NaOH mass to 0.8 g, the collected Fe_3_O_4_ nanoparticles exhibit an interesting multifaceted geometry (Fig. 1D,D’). High magnification TEM images further revealed that the majority of these multifaceted Fe_3_O_4_ nanoparticles are octahedrons (Fig. S1). Figure S2 shows the morphological uniformity of these magnetic nanostructures. The crystalline structure of the as-prepared Fe_3_O_4_ nanoparticles was characterized using X-ray diffraction (Fig. S3) and their magnetic property was evaluated using a vibrating sample magnetometer (VSM, see Fig. S4A).

In comparison to previously reported GO/Fe_3_O_4_ composites prepared through the *in situ* deposition of Fe_3_O_4_ nanoparticles, the GO/Fe_3_O_4_ nanohybrids reported here were obtained through simple yet efficient magnetic separation by taking advantage of the physical affinities between sulfonated GO and Fe_3_O_4_ nanoparticles. The fabrication route is schematically depicted in Fig. 1E (also see Methods). In the experiments, the pre-prepared Fe_3_O_4_ nanoparticles (either 20 or 40 mg) were dispersed in 40 mL of 1.25 mg·mL^−1^ GO suspension by ultrasonicating at high power. The GO/Fe_3_O_4_ nanohybrids were collected by magnetic separation to remove free floating GO, if any. The nanohybrid samples thus prepared were designated as G5F2 and G5F4, corresponding to the initial GO/Fe_3_O_4_ mass ratios of 5:2 and 5:4, respectively. Interestingly, it was found that the amount of free floating GO separated from the magnetic hybrids in a magnetic field was negligible. In addition to the physical affinities between Fe_3_O_4_ nanoparticles and the sulfonated GO sheets, which allow the nanoparticles to settle in-between GO layers, the flexibility and larger aspect ratio of the GO sheets used in this study can also allow Fe_3_O_4_ nanoparticles being wrapped by GO in their suspension, leading to highly efficient magnetic separation. The magnetic property of GO/Fe_3_O_4_ hybrids was shown in Fig. S4B. Figure 1B’ show the transmission electron micrographs of magnetic GO/Fe_3_O_4_ nanohybrids prepared from the Fe_3_O_4_ nanoparticles with different geometries, corresponding to Fig. 1B,C’, respectively.

**Figure 2:**
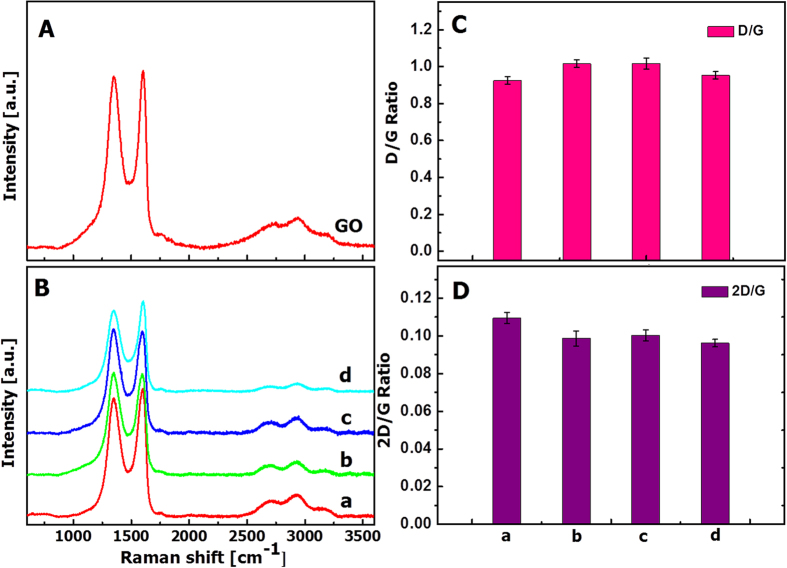
Raman spectra of lyophilized (**A**) GO and (**B**) hybrid composites. The intensity ratios of D/G and 2D/G in the Raman spectra shown in (**B**) were plotted in (**C**) and (**D**), respectively. [Note: Hybrid composites (**a**, **b**, **c**) were prepared with the Fe_3_O_4_ nanoparticles seen in Fig. 1B, C’, D’, respectively, using 5:4 by mass GO/Fe_3_O_4_. Hybrid composite (**d**) was prepared with purchased Fe_3_O_4_ nanoparticles using 5:4 by mass GO/Fe_3_O_4_].

Raman spectra, as shown in Fig. 2, were further collected to characterize the magnetic composite materials prepared in this study. Raman shift at 1601 cm^−1^, also called G band, was attributed to the first-order scattering of the E_2_g phonons of sp^2^-hybridized carbon atoms, while Raman shift at 1351 cm^−1^, i.e., D band ascribed to the breathing mode of the κ-point phonons of the A_1_g symmetry, originated from defects involved in sp^3^-hybridized carbon bonds (e.g., hydroxyl and epoxide bonds)^14^,^15^. In addition to the G and D bands, a broad peak at 2692 cm^−1^ (i.e., 2D band) was also observed. The intensity of 2D band is correlated to the stacking mode of graphene sheets^16^. Previous study has shown that, for single-layer graphene sheets, the G and 2D bands appear at 1585 and 2679 cm^−1^, respectively, while for multi-layer graphene sheets, both the G and 2D bands can shift in Raman spectra^17^,^18^, as shown in this study. Furthermore, the 2D/G intensity ratios for the single and bilayer GO sheets were found in the range of 1.53–1.68 and 0.82–0.89, respectively, as previously reported by O. Akhavan *et al*.^19^,^20^ Previous studies have also shown that the 2D/G intensity ratios for single-, double-, triple-, and multi- (>4) layer graphene sheets are 1.6, 0.8, 0.30 and 0.07, respectively^21^,^22^. Figure 2D shows that the 2D/G ratios of the GO sheets and four different composite samples prepared in this study have values between 0.09 and 0.11, further suggesting the multilayer nature of the GO-based materials. In addition, the G/D peak intensity ratio, known as a measure of the sp^2^ domain size of graphene sheets containing sp^3^ and sp^2^ bonds, only exhibits a negligible change for the GO-based magnetic composites in comparison with that of the neat GO sample (see Fig. 2C), implying that the property of the GO was not chemically altered during the preparation of the magnetic composites.

In the past years, the one-pot synthesis of GO-based nanocomposites through the *in situ* growth of nanostructures directly onto GO sheets has been developed into a state-of-art science subject^1^,^2^,^3^,^4^,^5^,^6^,^23^,^24^. Some of these GO-based nanocomposites have found their niches in potential applications as energy storage devices^25^, supercapacitors^26^, and electrode materials^27^. However, when the goal of a research task is to remove organic dyes by utilizing the superior adsorption capacity of a GO surface, the available surface area of GO sheets (i.e., the available surface area for π-π stacking interactions) along with the abundance of functional groups on the GO sheets becomes the most predominant factor in determining the dye removal efficiency. For instance, for GO/Fe_3_O_4_ nanocomposites containing *in situ* precipitated ultrafine nanoparticles (e.g., less than 50 nm diameter), the adsorption efficiency of pristine GO sheets could be decreased because (1) the Fe_3_O_4_ nanoparticles are permanently anchored on the GO sheets and (2) the ultrafine nanoparticles can occupy a greater surface area than relatively larger nanoparticles of the same total mass. Thus, the facile preparation of GO/Fe_3_O_4_ nanohybrids reported in this study was aimed at overcoming the above drawbacks by (1) combining the GO and Fe_3_O_4_ nanoparticles through relatively weak physical affinities and (2) tuning the dimensions and geometries of Fe_3_O_4_ nanoparticles to decrease the contact area with GO, while still maintaining the magnetic properties of the nanohybrids for easy separation and adsorbent recycling. The dye removal efficiency of the GO/Fe_3_O_4_ nanohybrids prepared in our study was evaluated thoroughly and the results are discussed below.

**Figure 3:**
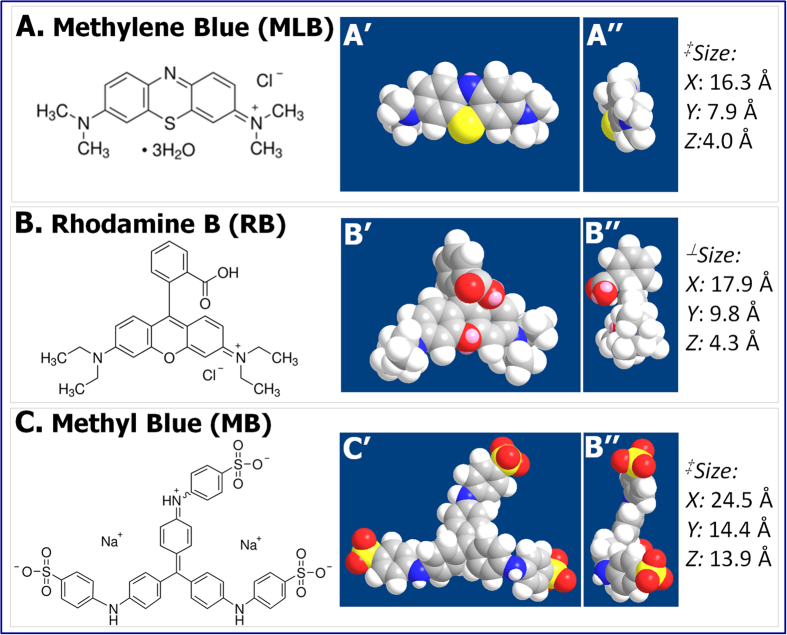
Chemical structures of (**A**)MLB, (**B**) RhB, and (**C**) MB, along with the top (**A**’, **B**’, and **C**’) and side (**A**”, **B**”, and **C**”) view of their space-filling models. [Note: ^‡^Ref. 29 and ^⊥^Ref. 30].

**Figure 4:**
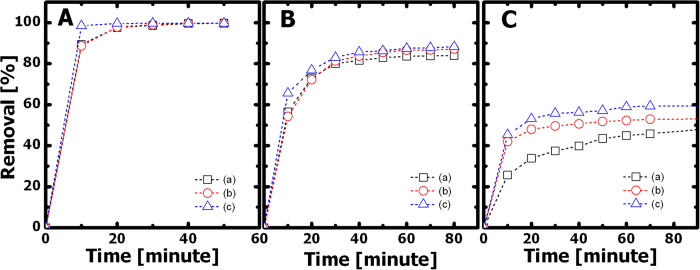
The percent dye removal rate versus time plots for the adsorption of (**A**) MLB, (**B**) RhB, and (**C**) MB using GO/Fe_3_O_4_ nanohybrids (G5F4) prepared from (**a**) fine nanoparticles, (**b**) nanospheres, and (**c**) multifaceted nanoparticles.

Two cationic dyes, methylene blue (MLB) and Rhodamine B (RhB), and one anionic dye, methyl blue (MB), were used in this study. Their molecular structures, space-filling models, and dimensions are shown in Fig. 3. Figure 4 shows the percent removal rate versus time plots for removing each dye in its aqueous solution (20 mg·L^−1^, 5 mL) using 3 mg of G5F4 nanohybrids prepared from (a) fine nanoparticles, (b) nanospheres, and (c) multifaceted nanoparticles. The percent dye removal rate was probed based on absorption spectroscopy (see Methods). Figure 4A shows an impressively high removal rate for MLB, which reaches above 95% within only 30 minutes, regardless of the geometry and dimensions of the incorporated Fe_3_O_4_ nanostructures. The adsorption behavior of RhB is also independent of the type of Fe_3_O_4_ nanostructures, as shown in Fig. 4B. The removal rate for RhB can also reach above 80% within just 30 minutes, though the rate is lower than that for MLB. In contrast, the adsorption rate for MB is found to vary with the dimensions of the Fe_3_O_4_ nanoparticles. For instance, the MB removal rate using GO/Fe_3_O_4_ nanohybrids containing finer nanoparticles (see morphology in Fig. 1B,B’) is less than 40% within the first 30 minutes, which is lower than the approximately 50% achieved by using nanohybrids containing larger nanospheres (see morphology in Fig. 1C-C”) and multifaceted nanoparticles (see morphology in Fig. 1D-D”). In addition, for MB solutions, the nanohybrids containing multifaceted nanoparticles did exhibit a slightly higher dye adsorption capacity than those prepared using larger nanospheres. Nevertheless, the GO/Fe_3_O_4_ nanohybrids thus prepared possess a much higher adsorption capacity for MLB and RhB than for MB, which can be attributed to the fact that (1) the electrostatic attractions between GO sheets and cationic dyes (i.e., MLB and RhB) are strong and (2) both the quality and quantity of π-π stacking interactions could decrease from MLB, to RhB, to MB, as suggested in Fig. 3. In Fig. 3, the side view of the space-filling models implies that the quality of molecular conjugation might decrease from MLB, to RhB, to MB, with the increasing dimensions of these dye molecules.

**Figure 5:**
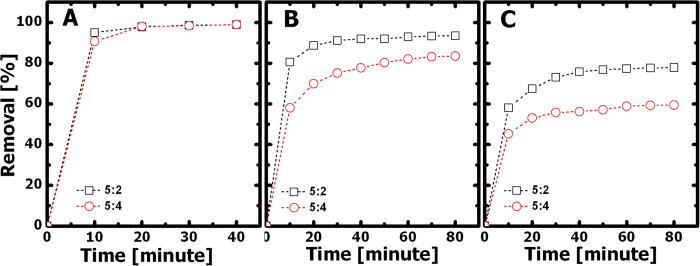
The percent dye removal rate versus time plots for the adsorption of (**A**) MLB, (**B**) RhB, and (**C**) MB using nanohybrids prepared with initial GO/Fe_3_O_4_ mass ratios of 5:2 (G5F2) and 5:4 (G5F4), respectively.

**Figure 6:**
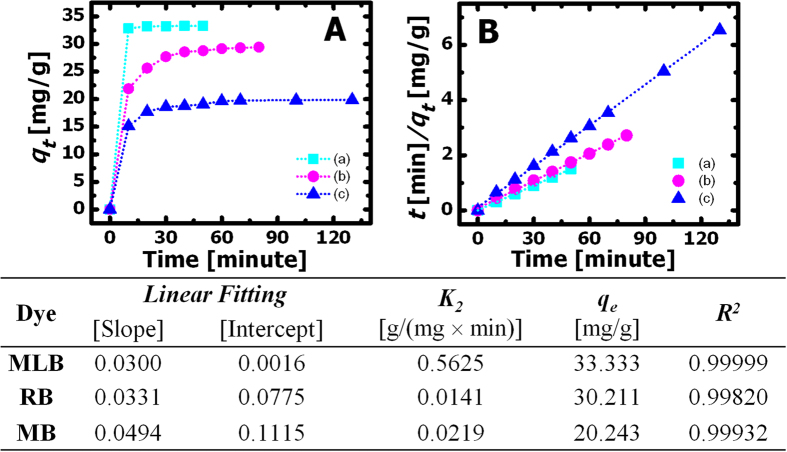
Kinetic adsorption (**A**) *q*_*t*_ versus *t* plots and (**B**) *t/q*_*t*_ versus *t* plots for (**a**) MLB, (**b**) RhB, and (**c**) MB. The table lists the fitting results achieved for kinetic adsorption data using the pseudo-second-order adsorption equation.

Figure 5 shows the percent dye removal rate versus time plots for the adsorption of (A) MLB, (B) RhB, and (C) MB, using nanohybrids with different GO to Fe_3_O_4_ mass ratios. [Note: The nanohybrids used here were prepared using the multifaceted nanoparticles, as seen in Fig. 1D,D’.] For MLB, the percent dye removal rate is comparable for both G5F2 and G5F4 samples and is nearly 100% within 30 minutes. In contrast, for both RhB and MB, when increasing the GO content in the nanohybrid samples, the dye adsorption capacity increases significantly, such as for the G5F2 sample, clearly indicating that it is the exposed GO surface area playing the determining role in the adsorption capacity of the GO/Fe_3_O_4_ nanohybrids. The above observation is in agreement with that found in rGO/Fe_3_O_4_ composites containing chemically anchored Fe_3_O_4_ nanoparticles^2^. For G5F4 samples prepared using multifaceted nanoparticles (Fig. 1C,C’), the adsorption kinetics were further evaluated by fitting experimental data using a pseudo-second-order adsorption equation: 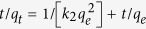, where *t* is the adsorption time, *q*_*e*_ is the adsorption capacity at equilibrium, *k*_*2*_ is the pseudo-second-order rate constant, and *q*_*t*_ is the adsorption capacity at time *t*^28^. The fitting results are summarized in a table in Fig. 6. In comparison to previous reports on RhB adsorption using GO or rGO-based composites with *in situ* deposited Fe_3_O_4_ nanoparticles^2^,^3^, the *q*_*e*_ value of 30 mg·g^−1^ is comparable to the reported values. Furthermore, the kinetic adsorption experiments using the G5F2 samples gave rise to an even greater *q*_*e*_ value of 32 mg·g^−1^. It is also worth mentioning that, in this study, both the compositions of the GO/Fe_3_O_4_ nanohybrids and other experimental conditions (e.g., pH, temperature, adsorption time, etc.) are not optimized for large scale applications. It is therefore reasonable to expect even higher *q*_*e*_ values after optimizing those experimental parameters that can potentially affect the adsorption capacity of the GO/Fe_3_O_4_ nanohybrids.

Extensive effort has been made to introduce permanently anchored Fe_3_O_4_ nanoparticles to GO or rGO sheets. The main goal of incorporating magnetic Fe_3_O_4_ nanoparticles in these studies, if not the only one, is to achieve rapid magnetic separation of dye-adsorbed composites from a water environment. However, as mentioned early, the functionalities and the available surface area of GO sheets are consequently compromised because of the anchored Fe_3_O_4_ nanoparticles. Moreover, it is fairly difficult to precisely control the composition of the final products synthesized through the one-pot, *in situ* deposition of Fe_3_O_4_ nanoparticles. This difficulty in compositional control could also prevent further large-scale industrial applications of these composites as adsorbents for dye or other organic pollutants. In sharp contrast, the Fe_3_O_4_ nanoparticles introduced in this study were only physically attached onto GO sheets and served mainly as magnetic agents for rapidly extracting dye-adsorbed GO sheets from an aqueous environment. Benefiting from both the rapid yet efficient magnetic separation and the weak affinities between sulfonated GO and Fe_3_O_4_ nanoparticles, the hybrid materials reported here can be easily regenerated.

**Figure 7:**
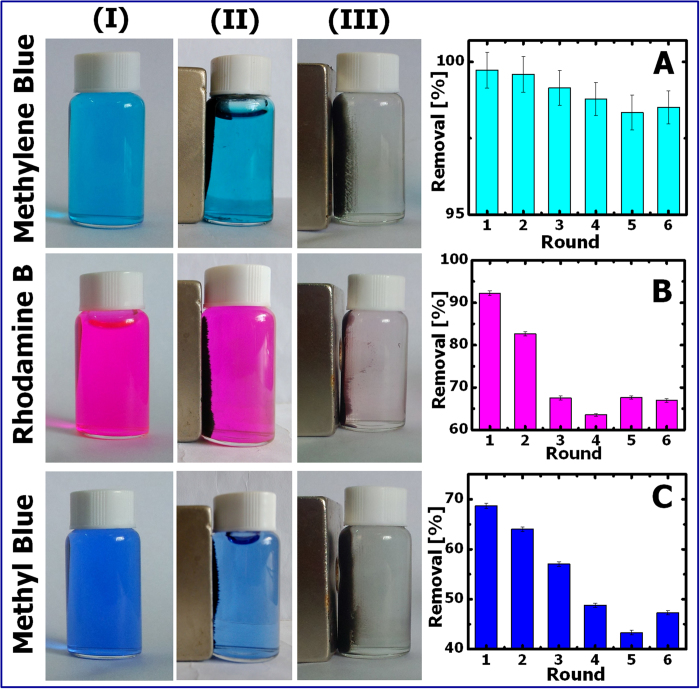
Photographs of dye solutions (20 mg·L^−1^) before (**I**) and after (**II**) adding Fe_3_O_4_ multifaceted nanoparticles or (**III**) adding GO/Fe_3_O_4_ nanohybrids. Percent dye removal rate was determined after 12-hour adsorption with constant shaking for the as-prepared GO/Fe_3_O_4_ nanohybrids (Round 1) and for the regenerated GO/Fe_3_O_4_ nanohybrids (Round 2–6), as seen in (**A**) MLB, and (**B**) RhB, and (**C**) MB.

In experiments, the percent dye removal rate acquired using an as-prepared G5F4 sample was determined after 12-hour adsorption with constant shaking and the value was designated as Round 1 (Fig. 7). The dye-adsorbed hybrid magnetically extracted from the aqueous solution was washed alternatively using ethylene glycol and ethanol several times, then washed with DI water, and finally, freeze-dried in a lyophilizer. It is also worth noting that the organic washing solvent turned to color solutions immediately after the dye molecules were released from the GO/Fe_3_O_4_ nanohybrids. This observation further suggests that the removal of organic dyes from aqueous solutions was achieved through the adsorption capacity of the prepared hybrid materials, which was also evidenced by the fitting results based on the pseudo-second-order adsorption kinetics, as discussed above. In addition, the Fe_3_O_4_ nanoparticles prepared here do not exhibit a photocatalytic effect on the degradation/removal of the three dyes (Fig. S5, S6, and S7), further demonstrating that the removal of the organic dyes discussed in this study is due to the high adsorption capacity of the GO/Fe_3_O_4_ hybrid composites. The regenerated hybrid composites were also used in the subsequent adsorption experiment, following the same procedure adopted in the 1^st^ Round experiment. The percent dye removal rate then determined was reported as the 2^nd^ Round result. For cationic MLB, the removal rate can reach nearly 100% using regenerated GO/Fe_3_O_4_ nanohybrids even after the 6^th^ Round (Fig. 7A), indicating that the recycling process hardly sacrifices the adsorption capacity of the hybrids for the MLB. For both RhB and MB, the percent removal rates decrease after each round of regeneration (Fig. 7B,C), suggesting that optimizing the hybrid compositions, adsorption conditions, and adsorbent recycling procedures is necessary to further improve the potency of regenerated GO/Fe_3_O_4_ hybrids as adsorbents for RhB and MB. Nevertheless, the percent dye removal rates for RhB can reach up to 90% (the 1^st^ Round) and 80% (the 2^nd^ round), which are still fairly high in comparison to previously reported values^2^,^3^.

## Conclusions

This study reports the facile preparation and the dye removal efficiency of GO/Fe_3_O_4_ nanohybrids. In comparison to previously reported GO/Fe_3_O_4_ composites prepared through the one-pot, *in situ* deposition of Fe_3_O_4_ nanoparticles, the GO/Fe_3_O_4_ nanohybrids reported here were obtained by taking advantage of the physical affinities between sulfonated GO and Fe_3_O_4_ nanoparticles. More importantly, since the Fe_3_O_4_ nanoparticles were prepared separately in this study, it was therefore possible to tune the dimensions and geometries of Fe_3_O_4_ nanoparticles to decrease their contact area with GO sheets, while still retaining their magnetic properties for easy separation and adsorbent recycling. For cationic MLB, the percent dye removal rate can reach nearly 100% by using regenerated GO/Fe_3_O_4_ hybrids even after the 6^th^ round, indicating the high potency of these hybrids. For cationic RhB, the percent removal rates can reach 90% in the 1^st^ round, 80% in the 2^nd^ round, and still above 60% in the 6^th^ round, which are comparable to or even better than previously reported results. This study provides new insights into the facile and controllable industrial scale fabrication of safe and highly efficient GO-based adsorbents for dye or other organic pollutants in a wide range of environmental-related applications.

## Methods

### Materials

Ferric chloride hexahydrate (FeCl_3_·6H_2_O, 98%), ethylene glycol, and graphite powder (200 mesh) were purchased from Tianjin Kaitong Chemicals (Tianjin, China). Rhodamine B, methylene blue, and methyl blue were obtained from Beijing Chemicals (analytical reagent grade, Beijing, China). All other solvents and reagents used in this study were purchased from Sinopharm Chemical Reagent Co. Ltd (analytical reagent grade, Shanghai, China). The deionized (DI) water was obtained using a Millpore Milli-Q water purification system with a resistivity of 18.2 MΩ cm^−1^. All chemicals were used as received without further purification.

### Synthesis of Fe_3_O_4_ Nanoparticles

Various Fe_3_O_4_ nanoparticles were prepared by a modified solvothermal method^13^. Ferric chloride hexahydrate (1.35 g) was first dissolved in 30 mL ethylene glycol, followed by adding sodium hydroxide (e.g., 0.4 g, 0.6 g, and 0.8 g) and polyethylene glycol (1.0 g) into the solution mixtures. After stirring for 30 min, the solution mixtures were transferred into a Teflon-lined stainless steel autoclave (100 ml) and reacted at 200 °C for 8 h. After the reaction was complete, the black precipitate (i.e., the Fe_3_O_4_ nanoparticles) was collected using an external magnetic field, followed by alternately washing with deionized water and ethanol several times. Finally, the Fe_3_O_4_ nanoparticles were dried at 60 °C in a vacuum oven.

### Preparation of Sulfonated GO Sheets

The GO sheets were prepared using a modified Hummer’s method^9^,^10^. Graphite, NaNO_3_, and concentrated H_2_SO_4_ were mixed together in a beaker in an ice bath for 30 min, followed by the slow addition of KMnO_4_. The reaction mixture was stirred at 35 °C for 6h, and then the temperature was slowly raised to 60 °C during the next 2h. The above mixture was then added to water and was stirred at 80 °C for 1 h, followed by adding 30% H_2_O_2_ and filtering. For purification, the product was alternately washed with 5% of HCl and then DI H_2_O several times. The filter cake was dissolved in DI H_2_O and the graphene oxide flakes were obtained through centrifugation. Finally, the product was freeze-dried in a lyophilizer for 2 days. In order to increase the capacity of uploading Fe_3_O_4_ nanoparticles, the GO sheets were sulfonated using a published method^12^. The prepared GO suspension (40 mL) was sulfonated with 98% sulfuric acid (10 mL) in an ice bath for 2 h, and washed with DI H_2_O several times to remove the remaining acid. The sulfonated GO sheets thus prepared were dried using a lyophilizer.

### Preparation of sulfonated GO/Fe_3_O_4_ Nanoparticle Hybrids

The sulfonated GO dispersion (50 mg GO in 40 mL water) was prepared by ultrasonicating GO in water for 1 h. A given amount of the Fe_3_O_4_ nanoparticles (e.g., 20 mg and 40 mg) was added to the GO aqueous dispersion. The mixture was then vigorously stirred for 30 min and ultrasonicated for 2 h to achieve a homogeneous suspension. The GO/Fe_3_O_4_ hybrids were collected by centrifuging and applying an external magnetic field to removeany floating GO sheets. Finally, GO/Fe_3_O_4_ hybrids were alternately washed with ethanol and DI H_2_O several times and dried using a lyophilizer.

### Dye Adsorption Experiments

The adsorption of dyes on the hybrid materials was examined using absorption spectroscopy. The prepared nanocomposites (3 mg) were added into a dye solution (20 mg L^−1^, 5 mL). The mixtures were ultrasonicated for 3 minutes and kept on a shaking table at 25 °C. The concentrations of residual dye in the aqueous solution were tested at different time intervals. The pH values of dye solutions were all adjusted to 6. This value was selected by optimizing the pH value for RhB (see Fig S8). A temperature of 25 °C and a pH value of 6 were then used for all absorption tests. Absorption values for a series of dye solutions with known concentrations were measured at the maximum absorption wavelength using a UV-vis spectrophotometer (554 nm for Rhodamine B, 664 nm for Methylene Blue, and 600 nm for Methyl Blue). Three calibration curves were therefore constructed in order to determine residual dye concentrations during adsorption experiments. The absorption value of the residual dyes was also determined at the maximum absorption wavelength using a UV-vis spectrophotometer.

### The Regeneration of the Hybrid Adsorbents

Once saturated adsorption for a given dye in an aqueous suspension was reached after 12 h on a shaking table at 25 °C, the hybrid was extracted from solution through an external magnetic attraction, and washed alternately using ethylene glycol and ethanol several times^3^. Finally, the regenerated hybrids were then washed with DI H_2_O and dried in a lyophilizer for subsequent adsorption experiments.

### Characterization

Scanning electron microscope (SEM) images were taken using a Hitachi S-4800 field emission scanning electron microscope (Chiyoda-ku, Japan) with an accelerating voltage of 5–15 kV. All transmission electron microscope (TEM) measurements were carried out using a Hitachi HT7700 transmission electron microscope (Hitachi, Tokyo, Japan). The X-ray diffraction (XRD) measurement was conducted using a Rigaku D/max 2550PC diffractometer (Rigaku Inc., Tokyo, Japan). The XRD pattern was obtained using CuKα radiation with an incident wavelength of 0.1542 nm under a voltage of 40 kV and a current of 200 mA. The scan rate was 0.5 degree·min^−1^. The magnetic properties of the samples were tested by a vibrating sample magnetometer (VSM) of the Physical Property Measurement System (PPMS, Quantum design Model 6000). The powders were aligned under a 4 kOe field and solidified with epoxy resin for the VSM measurements. Raman spectroscopy was performed using a Horiba Jobin Yvon Xplora PLUS confocal Raman microscope equipped with a motorized sample stage. The wavelength of the excitation laser was 532 nm and the power of the laser was kept below 1 mW without noticeable sample heating. The intensity of a Raman peak was extracted from the maximum value after baseline subtraction over corresponding spectral range.

## Additional Information

**How to cite this article**: Jiao, T. *et al*. Facile and Scalable Preparation of Graphene Oxide-Based Magnetic Hybrids for Fast and Highly Efficient Removal of Organic Dyes. *Sci. Rep*. **5**, 12451; doi: 10.1038/srep12451 (2015).

## Supplementary Material

Supplementary Information
