# Supplementary material for: Facile and Scalable Preparation of Graphene Oxide-Based Magnetic Hybrids for Fast and Highly Efficient Removal of Organic Dyes
Source: Sci Rep. 2015 Jul 29;5:12451. doi: 10.1038/srep12451 (PMC4518211; doi:10.1038/srep12451)
Supplement: Supplementary Information [file srep12451-s1.doc]

**Facile and Scalable Preparation of Graphene Oxide-Based Magnetic Hybrids for Fast and Highly Efficient Removal of Organic Dyes**

Tifeng Jiao^1,2,3^, Yazhou Liu^2^, Yitian Wu^2*^, Qingrui Zhang^2^, Xuehai Yan^4^, Faming Gao^2^, Adam J. P. Bauer^3^, Jianzhao Liu^3^, Tingying Zeng^5^, and Bingbing Li^3*^

^1^State Key Laboratory of Metastable Materials Science and Technology, Yanshan University, Qinhuangdao 066004, P. R. China

^2^Hebei Key Laboratory of Applied Chemistry, School of Environmental and Chemical Engineering, Yanshan University, Qinhuangdao 066004, P. R. China

^3^Department of Chemistry and Biochemistry, Central Michigan University, Mount Pleasant, MI 48859, USA

^4^National Key Laboratory of Biochemical Engineering, Institute of Process Engineering, Chinese Academy of Sciences, Beijing 100190, P. R. China

^5^Research Laboratory for Electronics, Massachusetts Institute of Technology, Cambridge, MA 02139, USA

*Corresponding Authors: Yitian Wu (wu6y@cmich.edu) and Bingbing Li (li3b@cmich.edu; 989-774-3441)


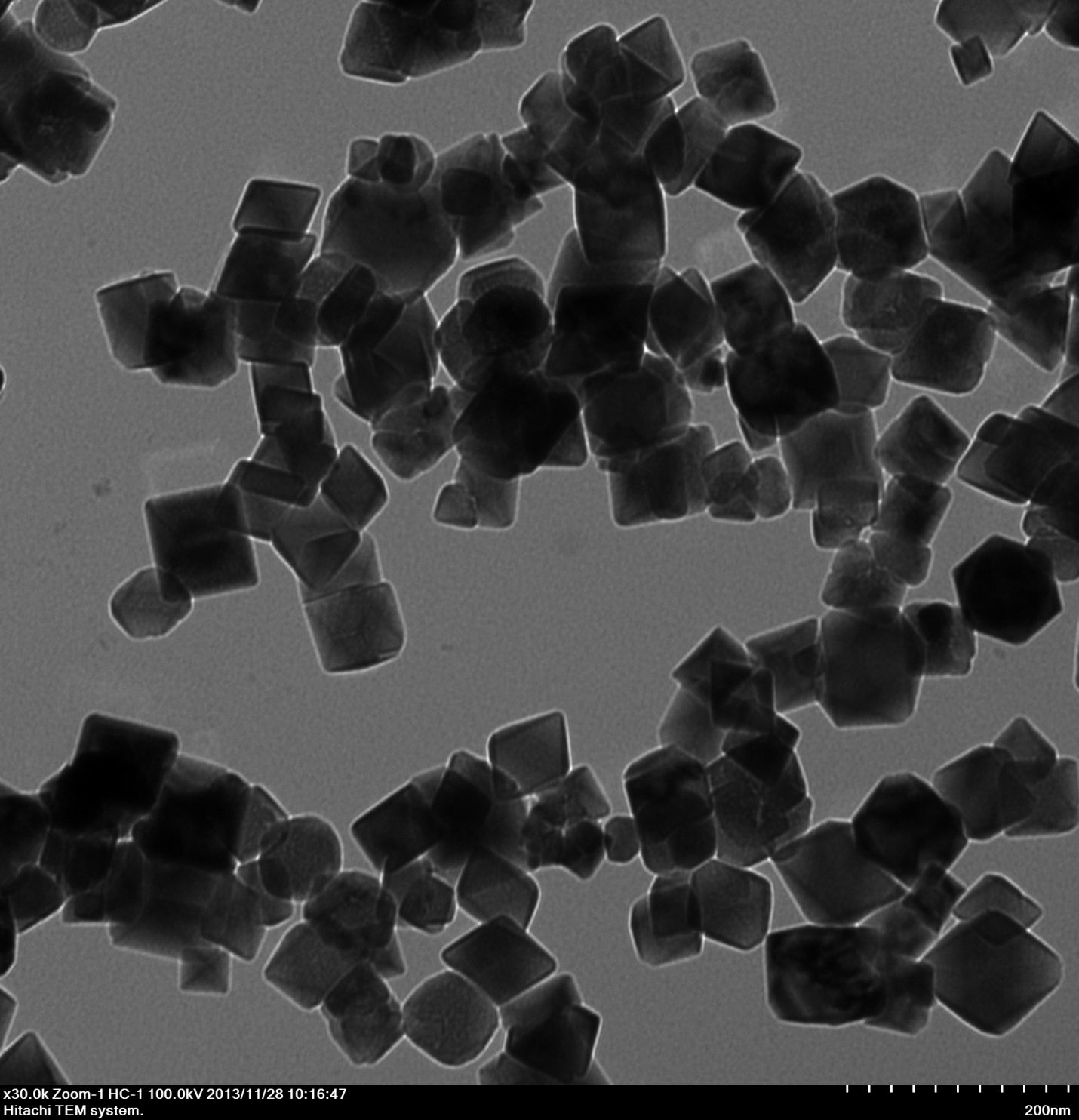
**Figure S1.** A TEM image of multifaceted Fe_3_O_4_ nanoparticles.

**Figure S2.** Morphology of Fe_3_O_4_ nanoparticles including (A, A’) fine nanoparticles (<100 nm diameter), (B, B’) nanospheres (~ 100 nm diameter), and (C, C’) multifaceted nanoparticles. Images A-C are scanning electron micrographs and images A’-C’ are transmission electron micrographs.

**Figure S3.** X-ray diffraction patterns for Fe_3_O_4_ nanoparticles prepared using different amount of sodium hydroxide (NaOH) (a) 0.4 g, (b) 0.6 g, and (c) 0.8 g, along with a purchased nanoparticle sample.

**Figure S4.** Magnetic hysteresis loops of (A) Fe_3_O_4_ nanoparticles and (B) 5:4 by mass GO/Fe_3_O_4_ hybrids. The Fe_3_O_4_ nanoparticles were prepared using different amount of sodium hydroxide (a) 0.4 g, (b) 0.6 g, and (c) 0.8 g.

**Figure S5.** The percent degradation rate versus time plots for methyl blue in the presence of neat Fe_3_O_4_ nanoparticles.

**Figure S6.** The percent degradation rate versus time plots for methylene blue in the presence of neat Fe_3_O_4_ nanoparticles.

**Figure S7.** The percent degradation rate versus time plots for Rhodamine B in the presence of neat Fe_3_O_4_ nanoparticles.

**Figure S8** The percent dye removal rate versus time plots for the adsorption of RhB at different pH values using GO/Fe_3_O_4_ nanohybrids (G5F2) prepared from multifaceted nanoparticles.
